# Practice guidelines for the supervising professional: intraoperative neurophysiological monitoring

**DOI:** 10.1007/s10877-018-0201-9

**Published:** 2018-10-30

**Authors:** Jeffrey H. Gertsch, Joseph J. Moreira, George R. Lee, John D. Hastings, Eva Ritzl, Matthew Allan Eccher, Bernard Allan Cohen, Jay L. Shils, Michael T. McCaffrey, Gene K. Balzer, Jeffrey R. Balzer, Willy Boucharel, Lanjun Guo, Leah L. Hanson, Laura B. Hemmer, Faisal R. Jahangiri, Jorge A. Mendez Vigil, Richard W. Vogel, Lawrence R. Wierzbowski, W. Bryan Wilent, James S. Zuccaro, Charles D. Yingling, the membership of the ASNM

**Affiliations:** 1 Univeristy of California San Diego School of Medicine, Department of Neurosciences, La Jolla, CA USA; 2 New York University/Winthrop University Hospital, Mineola, NY USA; 3 Vanderbilt University Medical Center, Department of Neurology, Nashville, TN USA; 4 Aeromedical Neurology, Jacksonville, FL USA; 5 Johns Hopkins University, Baltimore, MD USA; 6 Case Western Reserve University, Cleveland, OH USA; 7 Neurological Monitoring Associates, Milwaukee, WI USA; 8 Rush University Medical Center, Chicago, IL USA; 9 Michael McCaffrey Consulting, Sawyer, MI USA; 10 Real Time Neuromonitoring Associates, Nashville, TN USA; 11 University of Pittsburgh Medical Center, Pittsburgh, PA USA; 12 Children′s Hospital Colorado, Aurora, CO USA; 13 University of California San Francisco, San Francisco, CA USA; 14 Rhythmlink International, Columbia, SC USA; 15 Northwestern University Feinberg School of Medicine, Chicago, IL USA; 16 AXIS Neuromonitoring, Richardson, TX USA; 17 SpecialtyCare, Brentwood, TN USA; 18 SafePassage Neuromonitoring, New York, NY USA; 19 Avatrode, Bryn Mawr, PA USA; 20 Orlando Health, Orlando, FL USA; 21 Golden Gate Neuromonitoring, San Francisco, CA USA

**Keywords:** Intraoperative neurophysiology, Surgical neurophysiology, Intraoperative neurophysiological monitoring, Guideline, American Society of Neurophysiological Monitoring

## Abstract

The American Society of Neurophysiological Monitoring (ASNM) was founded in 1989 as the American Society of Evoked Potential Monitoring. From the beginning, the Society has been made up of physicians, doctoral degree holders, Technologists, and all those interested in furthering the profession. The Society changed its name to the ASNM and held its first Annual Meeting in 1990. It remains the largest worldwide organization dedicated solely to the scientifically-based advancement of intraoperative neurophysiology. The primary goal of the ASNM is to assure the quality of patient care during procedures monitoring the nervous system. This goal is accomplished primarily through programs in education, advocacy of basic and clinical research, and publication of guidelines, among other endeavors. The ASNM is committed to the development of medically sound and clinically relevant guidelines for the performance of intraoperative neurophysiology. Guidelines are formulated based on exhaustive literature review, recruitment of expert opinion, and broad consensus among ASNM membership. Input is likewise sought from sister societies and related constituencies. Adherence to a literature-based, formalized process characterizes the construction of all ASNM guidelines. The guidelines covering the Professional Practice of intraoperative neurophysiological monitoring were initially published January 24th, 2013, and subsequently that document has undergone review and revision to accommodate broad inter- and intra-societal feedback. This current version of the ASNM Professional Practice Guideline was fully approved for publication according to ASNM bylaws on February 22nd, 2018, and thus overwrites and supersedes the initial guideline.

## Introduction

Intraoperative neurophysiological monitoring (IONM) is the use of physiological monitoring techniques to assess neural integrity and/or to map or neuro-navigate within at-risk neural structures during various surgical procedures. This growing subspecialty field of medicine is supported by an expanding evidence base spanning nearly a half century [1–13]. The following IONM professional practice Guidelines have been established according to current broadly recognized principles, and directly apply to supervising practitioners who render IONM services in the United States of America [14–17]. An additional objective of these Guidelines is to provide the public with information about the roles and responsibilities of professionals in the execution of their supervisory duties.

These guidelines are based on the tenet that the delivery of IONM services by the Intraoperative Neuromonitoring Professional (IONM-P) constitutes a patient care activity; thus, important ethical mores guiding all medical professionals apply [18]. In practice, the IONM-P operates as a subspecialist clinical consultant in the operative environment, typically to provide a specific service during the primary procedure at the request of the surgeon. Hence while the surgeon and/or anesthesiologist will often prioritize patient care in the operative environment, the IONM-P has a duty to further contribute to patient safety and clinical care according to their unique expertise.

The delivery of IONM services by the IONM-P is currently provided (1) in-person in the operative suite, (2) via a remote/telemedicine model with services provided from outside of the operating room, and (3) a blend of both aforementioned models depending on patient care needs at the time. The telemedicine component of this framework has grown over time as an efficient and effective method of improving access to subspecialist IONM patient care. Furthermore, IONM service demand far exceeds the limited supply of qualified subspecialized providers, which often necessitates telemedicine oversight of multiple simultaneous cases [19–25]. The guidance here is applicable without regard to the physical location of the IONM-P. Recognizing this complex and evolving framework and its associated challenges, a set of core unified Guidelines is essential to promote best practice patterns [26].

The Guidelines are being released during an era of significant change and uncertain health care regulation and economics; therefore, no time horizon for full implementation is prescribed or suggested. Given rapidly evolving technologies and improvements in healthcare delivery systems, these Guidelines will be reviewed periodically to incorporate the latest information and science available.

## Abbreviations and definitions

ASNM: American Society of Neurophysiological Monitoring
EEG: Electroencephalography
EMG: Electromyography
EP: Evoked Potentials (i.e., somatosensory EPs, motor EPs, etc.)
IONM: Intraoperative neurophysiological monitoring
IONM-P: Intraoperative neurophysiological monitoring supervising professional
IONM-T: Intraoperative neurophysiological monitoring technologist
Physician: A physician with a license to practice medicine in the state where the surgical procedure is occurring
QHP: Qualified healthcare professional
Surgeon: An inclusive title used in this document to describe physicians performing the primary procedure monitored (i.e., traditional surgeons, neurovascular interventionalists, and other proceduralists)
Telemedicine: The remote delivery of health care services and clinical information using telecommunications technology [19, 20]

## Necessity for IONM-P practice guidelines

The IONM-P engages in IONM as a patient care activity whether or not the IONM-P is a physician or other qualified healthcare professional practitioner, and high ethical standards and obligations apply [18]. This document:

1. defines the scope of that patient care activity,
2. recommends ways to protect the interests of the patient,
3. delineates typical practice patterns for IONM-Ps at all levels of experience,
4. provides a coherent explanation of the IONM discipline to the larger universe of healthcare providers.

These Guidelines are intended to benefit hospital credentialing committees, state licensure boards, compliance regulators, commercial and governmental payers, medical and surgical societies, and others who request guidance on IONM standards of practice [26]. As a patient care activity, IONM practice differs significantly from neurophysiological laboratory studies (EEG, EPs, EMG, *etc*.). In the case of outpatient laboratory testing or other episodic neurophysiological procedures, the IONM-P is asked to identify possible neuropathophysiology after the testing has been performed. In contrast, during the course of IONM, the IONM-P is engaged in a real-time activity that requires the IONM-P to be continuously available to intervene or supervise IONM patient care contemporaneously during the entirety of the surgical procedure.

## IONM practice guidelines not a medicolegal document

These Guidelines are an attempt to define minimum IONM-P practices under typical circumstances. Because each surgical procedure has unique circumstances, a lack of adherence to some aspects of these Guidelines cannot be construed to imply negligence or breach of duty.

## IONM-P definition and qualifications [27]

### IONM-P definition

The IONM-P is the provider of real time technological supervision, interpretation, and diagnostic/therapeutic (interventional) suggestions or recommendations during IONM.

The ASNM recognizes, per the American Medical Association’s policy H-410.957, that the performance of IONM at a professional level (ie supervision, interpretation, and intervention in IONM) constitutes the practice of medicine [28]. The ASNM expects further guidance regarding roles, responsibilities, and qualifications for providers of IONM with the publication of “Guidelines for qualifications of neurodiagnostic personnel” by the Intersociety Neurodiagnostics Qualified Personnel Work Group; which includes the ASNM, American Clinical Neurophysiology Society (ACNS), American Association of Neuromuscular and Electrodiagnostic Medicine (AANEM), and American Society of Electroneurodiagnostic Technologists (ASET) (in draft stage as of this publication date).

### IONM-P qualifications

Each IONM-P must comply with local statutory authority concerning scope of practice and State licensure during delivery of patient care. Unless a given institution elects not to extend credentialing/privileges for IONM, the qualified IONM-P is always expected to maintain appropriate hospital credentialing and appropriate privileges at all facilities where they perform the duties of an IONM-P. Exceptions may be considered in the context of unexpected or emergent situations when a credentialed and privileged IONM-P is unavailable. When evaluating requests related to credentialing and privileging for IONM, hospitals are strongly encouraged to demand evidence of an appropriate combination of board certification or re-certification, training, experience, licensure (where applicable), and continuing education.

Board certification relevant to the practice of IONM patient care remains necessary to practice as an IONM-P, and must be secured within the board-eligible period as defined by the relevant board. In the absence of a time-based standard, the ASNM standard is 7 years for acquisition of board certification.

## IONM-P responsibilities: pre-operative

The IONM-P will provide oversight of the entirety of the IONM procedure as specified in their scope of practice. Additionally, the IONM-P will provide oversight of technical providers of IONM (or IONM-T), with the noteworthy caveat that the IONM-T carries responsibilities and liabilities as established in their respective scope of practice and guidelines (see below) [27, 29].

IONM patient care by the IONM-P includes evaluation of patient factors for IONM planning, intraoperative care including waveform interpretation, postoperative follow-up (as indicated), and the management of personnel who operate the instrumentation supporting these activities. In many instances (particularly a dedicated telemedicine model), the IONM-T will gather and present the data necessary for the IONM-P to provide a pre-operative patient assessment.

In order to achieve optimum patient care and safety, the IONM-P is responsible for the following:

### Management of personnel and instruments

The IONM-P often directs application of monitoring techniques performed by the supervised IONM-T [27, 30]. The IONM-P should be mindful of the skills and experience level of assigned IONM-T personnel for each patient and procedure. Electrical safety and HIPAA compliance of all instrumentation shall be tested and maintained by the appropriate technical, ‘biomedical’ service. The IONM-P or designated IONM-T confirms the proper functioning of all hardware, software programming, wired and wireless communication equipment, specialized stimulators, and recording leads to be used in IONM procedures. When necessary, any malfunctions or failures of hospital communication networks should be reported to the appropriate hospital personnel.

### Pre-IONM evaluation of patient information

A Pre-IONM evaluation of patient information permits for the development of a patient-specific IONM care plan that considers pre-operative clinical data points which may influence the results, interpretation, and reliability of IONM [31]. The content and extent of this evaluation is variable and dependent on patient needs, provider access, timing, and other factors. This evaluation may include, for example, review of the medical record for prior imaging and/or neurophysiological procedure results, history-taking, and a focused examination. The Pre-IONM evaluation is also an opportune time to provide the patient with a description of IONM procedures (and their risks) and gain assurance of informed decision making on the part of the patient. Additionally, whenever possible or as circumstances necessitate the IONM-P (or designated IONM-T) should interface with the care team, *i.e*., the:

1. Surgeon to identify specific operative goals and risks, to plan methods of assessing neural topography, to plan tactics in the event of an IONM signal change, or to discuss other relevant interests.
2. Anesthesiologist concerning the plan for induction and maintenance of anesthesia.

With due consideration of resources, feasibility and realities of implementation, Guideline authors recommend direct or indirect interaction with the patient to the extent possible. This can be accomplished through multiple pathways, including a collaboration with the IONM-T to gather necessary data and facilitate communications with the care team. Although the IONM-T may contribute significantly to the preoperative documentation of patient information and facilitate interaction with care team members, the IONM-P is ultimately responsible for the individualized evaluation.

### Prescribing the IONM plan

The IONM-P is responsible for formulating an individualized IONM patient care plan aimed at the greatest safety and highest quality of care for each patient. After consideration of patient information and care team communications, the IONM-P confirms the IONM strategy for a given case with the IONM-T. In some instances, the surgeon and/or anesthesiologist may assert their prerogative to alter the IONM plan as prescribed by the IONM-P in order to align and advance the overall goals of care.

## IONM-P responsibilities: conduct of IONM

### Intraoperative responsibilities

In order to optimize patient care and safety, the IONM-P must remain continuously available to perform intraoperative responsibilities in real time throughout the procedure. Retrospective, or ‘‘after-the-fact’’ interpretation and reporting, is of negligible benefit to the patient or the surgeon.

The IONM-P:

1. Supervises all technical aspects of IONM to ensure overall patient safety and quality of care.
2. Communicates and collaborates with other members of the patient care team as detailed in Sect. 7.1.1–7.1.4.
3. Interprets IONM data:
  - Evaluates the quality and consistency of baseline data and identifies abnormalities in the context of known variables.
  - Evaluates IONM data in the context of the procedure and takes into account patient vitals, imaging and labs when available and appropriate.
  - Evaluates and interprets data obtained from topographical/neuro-navigation studies.
4. Develops a differential diagnosis:
  - Determines the significance of changes from baseline data. To the extent possible, determines if changes are related to iatrogenic injury, anesthetic effects, physiological variables, patient positioning, technical factors, or a combination of these.
  - Recommends assessment technique(s) most appropriate to answer anatomic, functional, or prognostic questions related to specific neural structures.
5. Provides input to the patient care team to help develop and execute a plan of therapeutic intervention to recover neural function when an adverse alteration in IONM data presents.

#### Interactions with the IONM-T

The IONM-P:

1. Collaborates with the IONM-T to ensure that real-time communication is maintained throughout the monitored procedure. When the IONM-P is not in the operative suite, it is expected that a continuous, HIPAA-compliant feed of all live data being recorded will be available to the IONM-P for contemporaneous interpretation. In addition, at minimum, direct voice access (via ‘land-line’ or cellular network) for perioperative communication with the surgical team is expected.
2. Collaborates with the IONM-T to ensure that procedural context is communicated as needed to facilitate optimal data interpretation.
3. Collaborates with the IONM-T to optimize signal acquisition.
4. Directs neural topography/neuro-navigation methods as needed.
5. Directs all necessary communication with the surgeon and anesthesiologist. In the context of communication via telemedicine, the IONM-T may make a timely report of a waveform change to the other members of the patient care team in advance of receiving direction from the IONM-P.
6. Provides intraoperative education and mentoring regarding basic and applied intraoperative neurophysiology.

#### Interactions with the surgeon

The IONM-P:

1. Conveys to the surgeon baseline status of IONM data, including deficient or unobtainable data.
2. Conveys to the surgeon changes in IONM data as appropriate:
  - Changes in data of potentially iatrogenic origin are communicated to the surgical team as soon as possible, once reasonable suspicion of an impending neurological insult exists.
  - Changes in data of non-surgical origin are reported to the surgeon as needed and appropriate. This report will parallel attempts to further elucidate and correct the causative factor(s).
3. Consults with the surgeon directly upon request or as needed.

#### Interactions with the anesthesiologist (or other appropriate member of the anesthesiology team)

The IONM-P:

1. Conveys to the anesthesiologist what IONM modalities are planned, and advocates for maintenance of anesthetic conditions that optimize the likelihood of obtaining and maintaining high quality IONM data within the constraints of the patient’s physiology [32].
2. Communicates with anesthesia team regarding potential safety concerns specific to the utilization of IONM. In particular, recommends the use of bite blocks to safeguard against oral trauma when motor EPs are used. Recognizing that it is the ultimate responsibility of the anesthesiologist to manage the patient’s airway, the IONM-P understands that the decision as to whether or not to place bite blocks remains the prerogative of the anesthesiologist.
3. Changes in data are reported to the anesthesia team as needed and appropriate.
4. Requests, as needed, information from the anesthesia team regarding current physiologic parameters relevant to IONM.
5. Recognizes that the anesthesiologist has the primary responsibility to manage a complex array of physiological parameters and their fluctuations. Thus, the extent to which the anesthetic requirements of IONM can be met must be balanced with the obligation of the anesthesiologist to optimize overall perioperative patient safety.

#### Other IONM-P colleagues (in the transfer of care)

The IONM-P:

Takes steps to ensure the safe transfer of patient care. If IONM case responsibility must be transferred between professionals, a hand-off protocol should be followed. Examples of similar transfers of responsibility can be found in anesthesia practice and other clinical environments. Pertinent pre-operative clinical information, baseline studies, and intra-operative findings/complications, must be thoroughly communicated before completion of the responsibility transfer. The recipient of the transfer must be afforded the opportunity to ask questions.

#### IONM during concurrent cases; sole dedication of the IONM-P to IONM [22–27]

It is anticipated that the IONM-P may be responsible for oversight in concurrent surgical procedures (as may be the case with anesthesia care). The IONM-P must judge his or her maximum capacity based on the mix of case complexity and other factors such as connectivity for telemedicine providers. Sufficient attention must be apportioned to each case such that all duties of the IONM-P are maintained for all cases. It is further recognized that cases of greater complexity may require personal attendance in the operating room.

## Post-IONM follow-up

IONM-P patient care activities should not end with completion of surgery. Discussion with the surgical team or other patient care activities involving IONM may be indicated depending upon the circumstances. These activities may necessitate some level of clinical follow-up by the IONM-P.

## Documentation and reports

It is important to appropriately document and archive recorded IONM data during all monitored surgeries.

### IONM data recording and storage

Representative samples from the sequence of recorded IONM modalities should be archived in order to ensure that that the intraoperative course of that patient can be adequately reconstructed. All stimulus-evoked responses should be archived with any associated commentary, including triggered EMG and all EPs. Because most instruments of recent vintage permit analog to digital conversion of ‘live’ continuous tracings such as EEG and EMG, these tracings should be archived when possible; otherwise, frequent ‘‘screen saves’’ should be archived so that the case may be suitably reconstructed. ‘‘Screen saves’’ of representative pathologic discharges (alerts) and recurring artifacts should be archived so that the case may be thoroughly understood by any reviewer at a later time.

### Physiological parameters

Physiological parameters relevant to the case should be gathered by the IONM-T per appropriate technical guidelines and communicated on an ongoing basis to the IONM-P, and additionally as requested by the IONM-P [30].

### Report

The IONM-P should generate a report specific to the IONM session with (1) the patient, surgeon, and IONM care team identified, (2) the surgical procedure(s) performed, (3) the types of modalities recorded with a description of the baseline responses, (4) neural topographical/neuro-navigational data acquired (when applicable), (5) details of any significant changes in responses during the procedure, (6) interventional measures recommended (in the case of concern for injury), (7) final responses obtained, and (8) details of post-operative neurological status when possible and relevant.

## Education and quality assurance

IONM programs should provide ongoing education and quality assurance. The IONM-P needs to participate in and be aware of these activities. QA/QI programs for both the IONM-T and IONM-P should be established so that patient outcomes can be monitored over time.

## IONM protocols (IONM policy and procedure manual)

There are a number of sources that describe appropriate protocols for performing basic neurophysiological testing in the operating room. It is the responsibility of the IONM-P to synthesize and appropriately apply the recommendations set forth in these Guidelines as well as the peer reviewed literature in order to establish and maintain an IONM policy and procedure manual. Some helpful resources include:

1. American Clinical Neurophysiology Society (ACNS) Guidelines can be found at http://www.acns.org. Not every guideline has information specific to neurophysiological testing in the operating room but they do indicate good practices and are a valuable guide.
2. American Society for Neurophysiologic Monitoring (ASNM) Guidelines have been published for Somatosensory EPs, Auditory EPs, EMG/reflex studies, Motor EPs, Intra-Operative EEG, and Transcranial Doppler. These are valuable guides to best practices. Additional Guidelines will be made available in the future. ASNM Guidelines can be found on the ASNM website at: http://www.asnm.org/page/Guidelines.
3. American Society of Electroneurodiagnostic Technologists (ASET) guidelines and materials can be helpful for formulating technical guidelines, and may be found on their website at http://www.aset.org.

American Society of Neurophysiological Monitoring Statement on Clinical Practice Guidelines The American Society of Neurophysiological Monitoring (ASNM) periodically publishes Clinical Practice Guidelines consistent with the Institute of Medicine.^1^ These documents are defined consistent with the National Guidelines Clearinghouse^2^ guidance “Clinical practice guidelines are systematically developed statements to assist practitioner and patient decisions about appropriate health care for specific clinical circumstances.” The ASNM Guidelines Committee recognizes three possible approaches to the guideline-writing task: (1) an evidence based review, (2) a summary methodological review based on a national plebiscite, or (3) construction of a patient-centered method drawing upon the best aspects of the extant Intraoperative Neurophysiological Monitoring (IONM) practice models.

The ASNM Clinical Practice Guidelines are heterogeneous evidence-based clinical guidance documents; as in terminology utilized in describing and/or labeling the Clinical Practice Guidelines may use phrases “guideline”, “protocol”, “pathway” in different context by different guideline developers in an effort to allow the author’s most relevant terminology. The ASNM Clinical Practice Guidelines are eligible for publication regardless of the terminology label provided the Clinical Practice Guidelines are consistent with the ASNM Position Statements^3^ and key components recommended by Guidelines International Network.^4^

The ASNM Clinical Practice Guidelines recognize evolving technologies and clinical practice patterns warrant periodic review and update of the previously published Guidelines.

^1^Clinical Practice Guideline We Can Trust, Institute of Medicine (March, 2011).

^2^National Guidelines Clearinghouse, AHRQ, Agency for Healthcare Research and Quality.

^3^Morledge and Stecker, Journal of Clinical Monitoring and Computing (2006) 20: 43–46.

^4^Qaseem, et al. Ann Intern Med. (2012); 156: 525–531.
